# Supplementary material for: Association between oral microbiome and seven types of cancers in East Asian population: a two-sample Mendelian randomization analysis
Source: Front Mol Biosci. 2023 Nov 21;10:1327893. doi: 10.3389/fmolb.2023.1327893 (PMC10702768; doi:10.3389/fmolb.2023.1327893)
Supplement: Supplementary file 1 [file DataSheet1.ZIP › Supplementary Materials/MR plots for tongue/tongue═╝/Gastric cancer/pheno.887_to_gastric cancer_scatter.pdf]

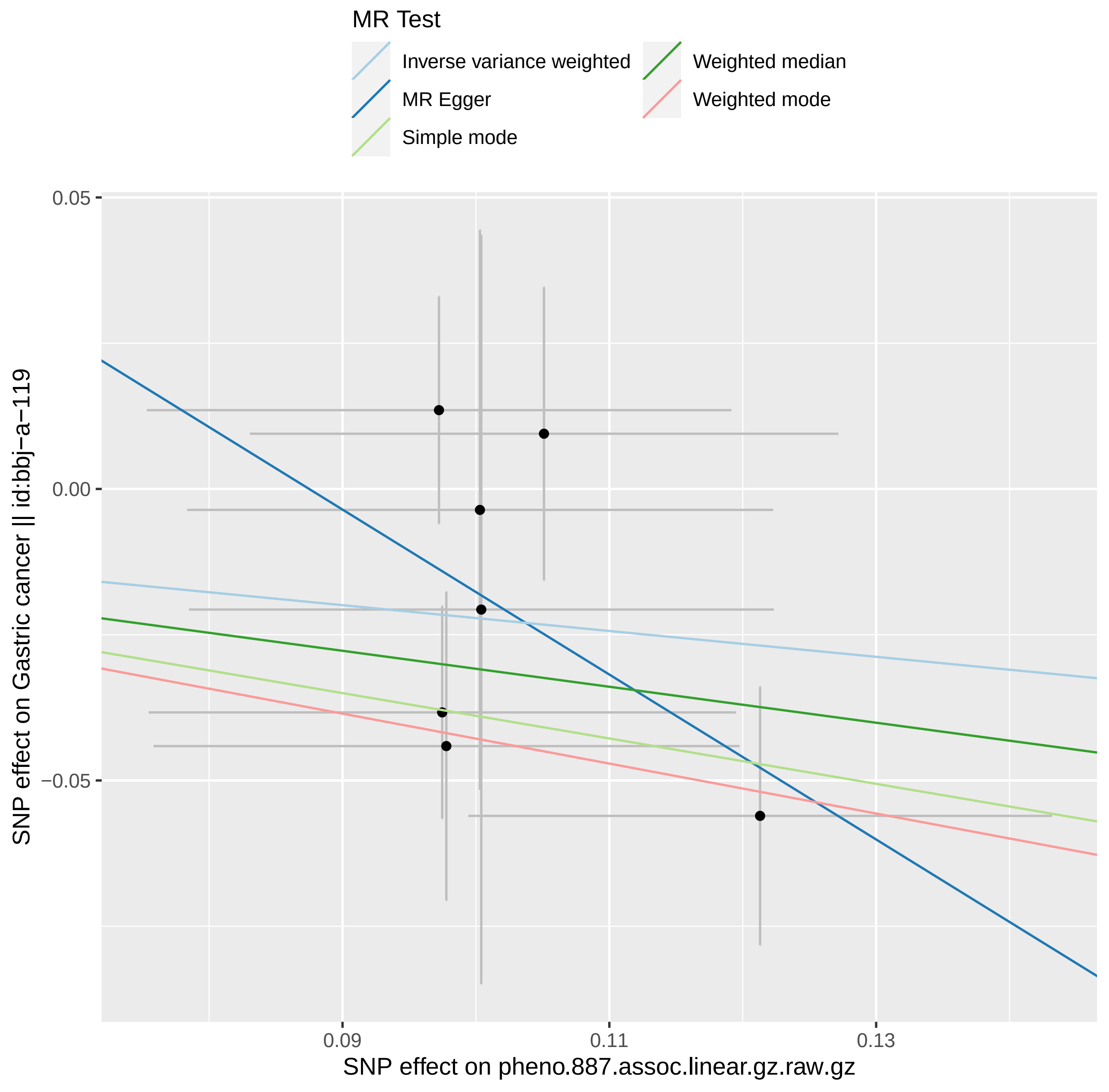

MR Test
Inverse variance weighted
MR Egger
Simple mode
Weighted median
Weighted mode
0.05
0.00
−0.05
0.09
0.11
0.13
SNP effect on pheno.887.assoc.linear.gz.raw.gz
SNP effect on Gastric cancer || id:bbj−a−119
